# Genome-wide identification and characterization of the HD-Zip gene family and expression analysis in response to stress in *Rehmannia glutinosa* Libosch

**DOI:** 10.1080/15592324.2022.2096787

**Published:** 2022-07-28

**Authors:** Yunhao Zhu, Shuping Peng, Le Zhao, Weisheng Feng, Chengming Dong

**Affiliations:** a School of Pharmacy, Henan University of Traditional Chinese Medicine, Zhengzhou, Henan, China; b Co-construction Collaborative Innovation Center for Chinese Medicine and Respiratory Diseases by Henan & Education Ministry of P.R.China, Zhengzhou, Henan, China

**Keywords:** HD-Zip transcription factors, *Rehmannia glutinosa* Libosch, bioinformatics analysis, biotic and abiotic stresses

## Abstract

The HD-Zip family of transcription factors is unique to the plant kingdom, and play roles in modulation of plant growth and response to environmental stresses. *R. glutinosa* is an important Chinese medicinal material. Its yield and quality are susceptible to various stresses. The HD-Zip transcription factors is unique to the plant, and roles in modulation of plant growth and response to environmental stresses. However, there is no relevant research on the HD-ZIP of *R. glutinosa*. In this study, 92 HD-Zip transcription factors were identified in *R. glutinosa*, and denominated as RgHDZ1-RgHDZ92. Members of RgHDZ were classified into four groups (HD-ZipI-IV) based on the phylogenetic relationship of *Arabidopsis* HD-Zip proteins, and each group contains 38, 18, 17, and 19 members, respectively. Expression analyses of *RgHDZ* genes based on transcriptome data showed that the expression of these genes could be induced by the endophytic fungus of *R. glutinosa*. Additionally, we showed that *RgHDZ* genes were differentially expressed in response to drought, waterlogging, temperature, and salinity treatments. This study provides important information for different expression patterns of stress-responsive HD-Zip and may contribute to the better understanding of the different responses of plants to biotic and abiotic stresses, and provide a molecular basis for the cultivation of resistant varieties of *R. glutinosa*.

## 1. Introduction

*R. glutinosa* and its active principles possess wide pharmacological actions on the blood system, immune system, endocrine system, cardiovascular system, and the nervous system. *R. glutinosa* yield and quality are limited by biotic, such as root rot, wheel spot, and fusarium wilt caused by microorganisms, as well as abiotic stresses, such as salt, heavy drought, and waterlogging. Exploring the molecular foundation of the gene-regulatory systems underlying plant responses to both abiotic and biotic stresses is crucial for medicinal plants improvement. In this regard, homeodomain leucine zipper (HD-Zip) genes family concerned with enlightening plant growth and tolerance to environmental stresses are considered key players for plants improvement.^1^ Therefore, excavation stress-related genes can provide a theoretical basis for breeding *R. glutinosa* resistant varieties. Ma Ligang et al. reported a reference genome of *R. glutinosa* using Nanopore technology, Illumina, and Hi-C sequencing. The assembly genome is 2.49 Gb long with a scaffold N50 length of 70 Mb and high heterozygosity (2%).^2^This laid the foundation for the discovery of genes related to *R. glutinosa* stress. Transcription factors can specifically bind to specific sequences of target genes, activate, or inhibit the expression of target genes, and then regulate a variety of life processes. The HD-Zip transcription factors is unique to the plant, and roles in modulation of plant growth and response to environmental stresses.^3^ It has been identified in a variety of plants, such as 33 in grapes (*Vitis vinifera* L.),^4^ 55 in corn,^5^ 48 in *Arabidopsis thalian*a,^5^ 45 in sesame,^3^ and 63 in *Populus trichocarpa*.^6^

HD-ZIP family transcription factors have two domains, homeodomian (HD) and Leucine-Zip (LZ). The HD is 60 amino acids in length and adopts a structure of three α-helices connected by a loop and a turn. The leucine-zipper is formed by five or more highly conserved leucine residues and, in general, exactly located every six amino acids. The HD-Zip family further divided into four subfamilies, HD-ZipI-IV, according to the conserved HD-Zip domain, additional conserved domain, structure features, and functions. HD-Zip class I protein has the simplest structure, which is closely related to plant abiotic stress, ABA response, blue light, de-etiolated, and embryogenesis.^7,8^ Overexpression of MdHB-7 promoted endogenous ABA accumulation, stomatal closure, and reactive oxygen species (ROS) detoxification in response to drought, whereas suppression of MdHB-7 expression had the opposite effect.^9^ A quantitative real-time PCR analysis indicated *Phehdz1*, a HD-Zip of Phyllostachys edulis expression was significantly induced by drought, high salinity, and abscisic acid (ABA).^10^ HD-ZipII TFs have been documented to affect plant growth and development mainly by regulating auxin, and have a distinguishing feature in their C-terminus, the CPSCE motif responsible for redox regulation of protein activity.^11–14^ In addition, the HD-ZIPII genes also responds to biotic and abiotic stress in plants.^15,16^ HD-ZipIII proteins can be distinguished from HD-ZipIV proteins by the presence of C-terminal MEKHLA domain which is absent in the HD-ZipIV proteins.^17^ HD-ZipIII TFs are mainly involved in the formation of plant vascular bundles, auxin transport,^18^ and organ development.^19–21^ In addition, they are also involved in plant abiotic stress tolerance, such as OsHB4 modulates cadmium tolerance and accumulation in rice. HD-ZipⅣ TFs play vital role in the development of plant epidermis.^22,23^ Cai found that the HD-ZipIV transcription factor GL2-LIKE regulates male flowering time and fertility in cucumber.^24^ In addition to regulating epidermal development, HD-ZipIV is also involved in plant abiotic stress response.^25^

In recent years, miRNAs have been noticeable with their important biological functions. MicroRNAs (miRNAs), a class of endogenous small regulatory non-coding RNAs, are usually 20–24 nucleotides long that play pivotal roles in gene regulation during plant development.^26,27^ Studies have shown that HD-ZipIII subfamily TFs are regulated by miRNAs to affect plant growth and development and resisitance.^28,^^29,30^ HD-ZipIII genes are the main target genes of miR166, which have been down-regulated by miR166 to regulate the response of tomato seedlings to cold stress.^31^ MiR166 plays a role in biotic and abiotic stress responses by regulating the transcription level of HD-ZipIII in *Arabidopsi*s.^32^ miRNAs are able to cleave their target mRNA of HD-Zip genes, thus regulating the functions of these genes. Therefore, predicting the miRNA that regulates HD-Zip genes is the important method for studying biological functions of HD-Zip TFs.

At present, there have been no relevant research on the HD-Zip transcription factors of *R. glutinosa*. In this study, *R. glutinosa* was used as the material to identify and explore the functions of HD-Zip genes. It provided a theoretical basis for revealing the anti-adversity regulation mechanism of *R. glutinosa*, which through qRT-PCR analysis, we explored the response of *RgHDZ* to different abiotic stresses, deepened our understanding of *RgHDZ*.

## 2. Results

### 2.1. Physicochemical properties and structure analysis of R. glutinosa HD-Zip transcription factor

According to HMMER search, a total of 92 non-redundant *R. glutinosa* HD-Zip encoding genes were identified denominated as RgHDZ1-RgHDZ92. *RgHDZs* encode proteins of 163–858 amino acids, and the molecular weight of RgHDZ is 18.99kDa-93.91 kDa. Except for Class II HD-Zips TFs, the isoelectric points of most proteins of other subfamily are less than 7. It was indicated that Class II is composed of more basic amino acids, while other proteins contain more acidic amino acids. In addition, the average hydrophobicity of RgHDZ proteins is less than 0, indicating that they are all hydrophilic proteins (Table 1). Subcellular localization analysis revealed that RgHDZ proteins are located in the nucleus (Supplementary Table 1). Transmembrane analysis showed that RgHDZ proteins are non-transmembrane protein.

**Table 1.** Physical and chemical properties and secondary structure prediction of RgHDZ proteins.

class	Gene name	GeneID	Len /aa	Mw/KDa	pI	Average hydropho-bicity	Prediction of Protein secondary structure			
							α- helix/%	extension chain/%	β-turn/%	coil/%
I	RgHDZ1	Rgl0091950.1	202	23.37	8.84	−0.97	47.29%	9.36%	2.46%	40.89%
I	RgHDZ2	Rgl0263140.1	201	23.49	6.82	−0.89	45.27%	6.97%	1.99%	45.77%
I	RgHDZ3	Rgl0259050.1	206	24.01	7.22	−0.86	43.90%	13.17%	4.88%	38.05%
I	RgHDZ4	Rgl0484050.1	181	20.92	4.72	−1.09	44.44%	5.00%	4.44%	46.11%
I	RgHDZ5	Rgl0290730.1	321	36.80	5.68	−0.61	35.51%	14.33%	3.43%	46.73%
I	RgHDZ6	Rgl0075610.1	310	35.85	4.79	−0.92	29.03%	11.29%	2.26%	57.42%
I	RgHDZ7	Rgl0489130.1	306	34.76	4.54	−0.79	33.01%	11.11%	2.61%	53.27%
I	RgHDZ8	Rgl0542770.1	264	29.90	6.15	−0.59	34.60%	13.69%	2.66%	49.05%
I	RgHDZ9	Rgl0366680.1	282	31.89	4.63	−0.59	27.30%	14.89%	2.48%	55.32%
I	RgHDZ10	Rgl0061960.1	310	35.63	4.84	−0.74	36.13%	9.68%	1.94%	52.26%
I	RgHDZ11	Rgl0501090.1	235	26.88	4.50	−1.01	43.83%	8.94%	4.26%	42.98%
I	RgHDZ12	Rgl0313970.1	270	30.60	4.29	−0.94	43.49%	13.01%	5.95%	37.55%
I	RgHDZ13	Rgl0083550.1	178	21.01	6.81	−1.18	53.11%	5.65%	5.08%	36.16%
I	RgHDZ14	Rgl0041480.1	207	24.34	5.48	−1.10	46.38%	9.18%	2.42%	42.03%
I	RgHDZ15	Rgl0349550.1	208	24.34	5.27	−1.10	46.38%	9.18%	2.42%	42.03%
I	RgHDZ16	Rgl0276970.1	250	28.48	6.01	−0.86	34.40%	2.40%	1.20%	62.00%
I	RgHDZ17	Rgl0113930.1	250	28.36	6.40	−0.84	40.96%	6.02%	1.61%	51.41%
I	RgHDZ18	Rgl0318420.1	247	28.19	5.86	−0.84	39.27%	4.45%	1.62%	54.66%
I	RgHDZ19	Rgl0365300.1	245	28.24	6.52	−0.77	34.84%	9.43%	3.69%	52.05%
I	RgHDZ20	Rgl0075480.1	241	27.77	6.15	−0.90	33.61%	5.81%	4.56%	56.02%
I	RgHDZ21	Rgl0044170.1	221	25.28	7.60	−1.04	40.45%	2.27%	2.27%	55.00%
I	RgHDZ22	Rgl0188480.1	165	19.13	9.60	−0.78	50.00%	3.05%	1.83%	45.12%
I	RgHDZ23	Rgl0393690.1	311	35.53	5.17	−0.96	36.01%	8.68%	2.25%	53.05%
I	RgHDZ24	Rgl0358730.1	332	37.71	5.47	−1.05	40.36%	10.84%	3.31%	45.48%
I	RgHDZ25	Rgl0158180.1	305	34.92	4.75	−0.81	31.80%	10.82%	3.28%	54.10%
I	RgHDZ26	Rgl0081860.1	306	34.83	4.56	−0.82	37.38%	8.52%	2.30%	51.80%
I	RgHDZ27	Rgl0178480.1	234	27.05	7.68	−0.78	36.32%	10.26%	2.56%	50.85%
I	RgHDZ28	Rgl0174710.1	235	27.07	8.15	−0.76	35.47%	9.83%	2.99%	51.71%
I	RgHDZ29	Rgl0029030.1	188	21.78	8.94	−0.95	48.40%	12.23%	4.26%	35.11%
I	RgHDZ30	Rgl0186960.1	181	20.71	8.34	−0.99	43.65%	7.73%	4.42%	44.20%
I	RgHDZ31	Rgl0324170.1	200	23.13	7.72	−0.95	43.50%	10.00%	2.50%	44.00%
I	RgHDZ32	Rgl0177210.1	201	23.28	7.73	−0.99	34.33%	10.95%	4.98%	49.75%
I	RgHDZ33	Rgl0124030.1	186	21.55	6.35	−0.83	44.62%	11.29%	4.30%	39.78%
I	RgHDZ34	Rgl0039560.1	169	19.38	7.25	−0.64	45.24%	13.10%	2.98%	38.69%
I	RgHDZ35	Rgl0525630.1	213	23.71	5.06	−0.36	43.19%	10.33%	7.04%	39.44%
I	RgHDZ36	Rgl0209560.1	191	21.25	5.96	0.58	41.36%	9.42%	5.76%	43.46%
I	RgHDZ37	Rgl0233540.1	198	22.06	8.25	−0.63	48.99%	4.04%	5.56%	41.41%
I	RgHDZ38	Rgl0363880.1	190	21.21	5.96	−0.60	44.74%	6.32%	4.21%	44.74%
II	RgHDZ39	Rgl0373580.1	259	29.01	8.60	−0.73	34.75%	11.20%	1.16%	52.90%
II	RgHDZ40	Rgl0003650.1	260	29.11	8.67	−0.75	28.57%	12.74%	1.93%	56.76%
II	RgHDZ41	Rgl0384520.1	211	24.04	9.58	−0.75	38.10%	16.67%	2.86%	42.38%
II	RgHDZ42	Rgl0420890.1	262	28.87	8.06	−0.69	27.97%	11.49%	1.53%	59.00%
II	RgHDZ43	Rgl0425010.1	291	32.62	7.56	−0.71	26.80%	8.25%	1.03%	63.92%
II	RgHDZ44	Rgl0108510.1	252	28.11	7.85	−0.80	28.29%	13.15%	1.59%	56.97%
II	RgHDZ45	Rgl0493760.1	250	28.00	8.53	−0.78	34.00%	12.00%	2.00%	52.00%
II	RgHDZ46	Rgl0039900.1	386	43.65	8.91	−0.91	24.68%	14.81%	2.60%	57.92%
II	RgHDZ47	Rgl0520310.1	380	41.86	6.12	−0.87	20.58%	12.14%	2.11%	65.17%
II	RgHDZ48	Rgl0363280.1	294	33.29	8.90	−0.76	26.53%	12.24%	3.06%	58.16%
II	RgHDZ49	Rgl0472170.1	316	35.42	8.45	−0.78	21.20%	17.09%	3.48%	58.23%
II	RgHDZ50	Rgl0196240.1	284	31.85	9.00	−0.90	28.62%	13.78%	2.12%	55.48%
II	RgHDZ51	Rgl0002900.1	216	24.35	8.92	−0.91	40.74%	11.57%	2.78%	44.91%
II	RgHDZ52	Rgl0008910.1	218	24.56	8.60	−0.90	33.18%	11.98%	3.69%	51.15%
II	RgHDZ53	Rgl0507440.1	236	27.08	7.06	−0.62	31.36%	13.14%	5.93%	49.58%
II	RgHDZ54	Rgl0332550.1	163	19.00	8.77	−0.87	49.69%	9.82%	3.07%	37.42%
II	RgHDZ55	Rgl0107540.1	264	30.01	8.48	−0.82	36.74%	14.39%	4.92%	43.94%
II	RgHDZ56	Rgl0070080.1	170	19.85	8.12	−1.07	43.20%	10.06%	1.18%	45.56%
III	RgHDZ57	Rgl0221390.1	829	91.38	6.55	−0.14	41.18%	15.22%	4.95%	38.65%
III	RgHDZ58	Rgl0129510.1	813	89.78	6.63	−0.17	44.46%	13.42%	4.80%	37.32%
III	RgHDZ59	Rgl0539910.1	836	91.71	6.22	−0.23	43.47%	14.25%	4.43%	37.84%
III	RgHDZ60	Rgl0449060.1	791	87.17	6.28	−0.17	43.24%	14.92%	4.05%	37.80%
III	RgHDZ61	Rgl0169870.1	766	83.79	7.44	−0.12	39.74%	15.29%	6.54%	38.43%
III	RgHDZ62	Rgl0136510.1	836	91.69	6.53	−0.19	39.35%	15.91%	5.26%	39.47%
III	RgHDZ63	Rgl0216740.1	858	93.91	7.13	−0.15	42.12%	15.17%	3.85%	38.86%
III	RgHDZ64	Rgl0467500.1	775	85.13	6.54	−0.12	43.80%	14.73%	4.26%	37.21%
III	RgHDZ65	Rgl0180880.1	803	88.16	6.39	−0.13	41.52%	15.59%	4.11%	38.78%
III	RgHDZ66	Rgl0085400.1	785	86.25	6.02	−0.12	39.29%	15.56%	5.48%	39.67%
III	RgHDZ67	Rgl0157430.1	816	88.70	6.11	−0.13	42.21%	14.48%	6.13%	37.18%
III	RgHDZ68	Rgl0435640.1	790	86.29	5.99	−0.07	42.66%	14.18%	5.19%	37.97%
III	RgHDZ69	Rgl0110560.1	812	88.51	5.97	−0.06	41.80%	14.80%	4.44%	38.96%
III	RgHDZ70	Rgl0342930.1	825	90.19	6.11	−0.11	39.76%	14.91%	5.45%	39.88%
III	RgHDZ71	Rgl0215380.1	813	89.61	6.78	−0.14	42.00%	13.42%	6.16%	38.42%
III	RgHDZ72	Rgl0266530.1	729	79.97	6.02	−0.13	42.94%	13.31%	5.62%	38.13%
III	RgHDZ73	Rgl0285320.1	756	83.11	6.33	−0.14	40.93%	14.83%	6.36%	37.88%
IV	RgHDZ74	Rgl0372770.1	657	74.02	6.75	−0.32	34.40%	15.37%	4.41%	45.81%
IV	RgHDZ75	Rgl0093910.1	631	70.41	6.32	−0.33	32.86%	16.83%	4.44%	45.87%
IV	RgHDZ76	Rgl0136710.1	712	80.31	6.33	−0.50	37.78%	16.85%	4.92%	40.45%
IV	RgHDZ77	Rgl0236930.1	725	79.98	6.04	−0.41	36.97%	14.21%	3.86%	44.97%
IV	RgHDZ78	Rgl0197090.1	615	68.62	6.58	−0.45	38.44%	14.17%	3.75%	43.65%
IV	RgHDZ79	Rgl0088000.1	742	82.43	4.93	−0.39	34.95%	17.54%	4.05%	43.45%
IV	RgHDZ80	Rgl0181610.1	668	74.10	6.5	−0.44	39.37%	13.62%	3.59%	43.41%
IV	RgHDZ81	Rgl0439490.1	671	73.45	5.88	−0.38	39.05%	13.11%	4.77%	43.07%
IV	RgHDZ82	Rgl0082500.1	696	75.86	6.18	−0.34	35.83%	14.39%	4.32%	45.47%
IV	RgHDZ83	Rgl0459290.1	626	69.84	6.31	−0.50	41.69%	12.30%	3.35%	42.65%
IV	RgHDZ84	Rgl0049310.1	651	71.16	5.76	−0.37	38.77%	12.31%	3.38%	45.54%
IV	RgHDZ85	Rgl0445290.1	667	72.72	6.63	−0.28	34.63%	16.04%	4.20%	45.13%
IV	RgHDZ86	Rgl0482470.1	782	85.52	5.82	−0.26	35.81%	13.68%	4.60%	45.91%
IV	RgHDZ87	Rgl0388660.1	782	85.37	6.15	−0.25	35.21%	13.44%	3.97%	47.38%
IV	RgHDZ88	Rgl0395390.1	602	66.23	5.81	−0.27	38.21%	13.62%	5.98%	42.19%
IV	RgHDZ89	Rgl0543870.1	635	69.57	7.92	−0.45	33.23%	14.33%	4.09%	48.35%
IV	RgHDZ90	Rgl0428480.1	784	85.54	6.16	−0.33	31.42%	15.45%	4.60%	48.53%
IV	RgHDZ91	Rgl0532970.1	656	72.79	6.31	−0.31	38.02%	15.73%	4.89%	41.37%
IV	RgHDZ92	Rgl0204940.1	754	82.49	6.15	−0.33	34.13%	14.48%	4.38%	47.01%

### 2.2. Phylogenetic analysis amongst the R. glutinosa

**Figure 1.**
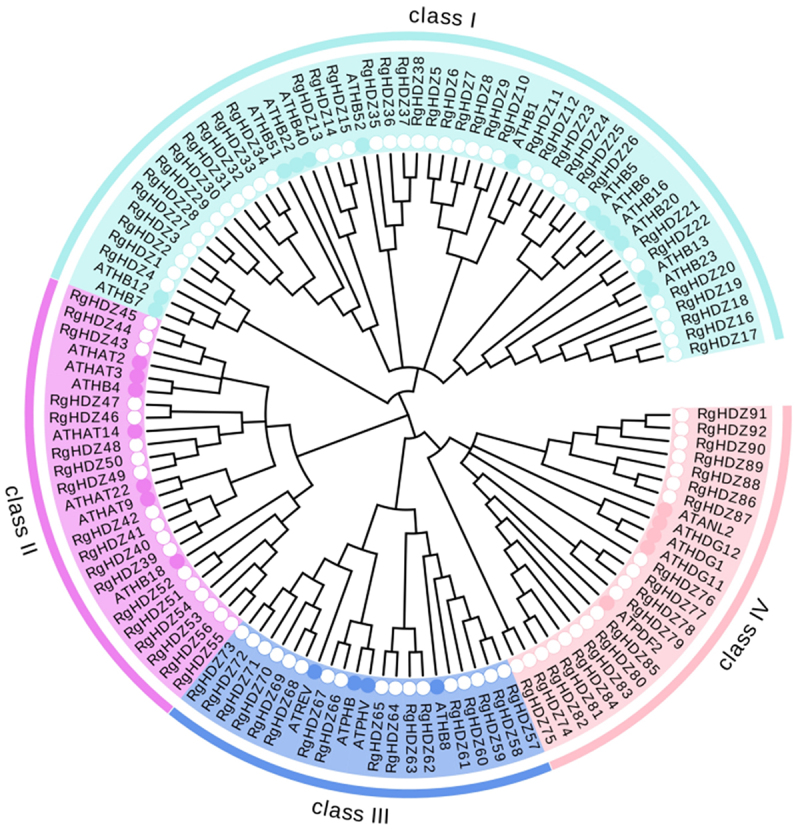
Phylogenetic tree of HD-Zip genes in *Rehmannia glutinosa* and *Arabidopsis thaliana*. The different-colored arcs indicate different groups of HD-Zip proteins. The solid circles and hollow circles represent HD-Zip proteins from Arabidopsis thaliana and Rehmannia glutinosa, respectively. HD-Zip proteins from Arabidopsis with the prefix ‘AT’. The phylogenetic tree was drawn using MEGA 7.

To reveal the phylogenetic relationships among the RgHDZ proteins, an unrooted phylogenetic tree was created to assess the genetic relationships between *Arabidopsis* and *R. glutinosa* HD-Zip proteins. The sequences of HD-Zip proteins of *Arabidopsis* were obtained from NCBI. As shown in Figure 1, these proteins can be divided into four distinct groups (HD-ZipI-IV), which is similar to that described in previous studies. Number of HD-ZipI-IV members in *R. glutinosa*was 38, 18, 17 and 19 respectively (Figure 1). The results provide an important basis for functional prediction of HD-Zip proteins in *R. glutinosa*.

### 2.3. Motifs and chromosome localization analysis of RgHDZs

**Figure 2.**
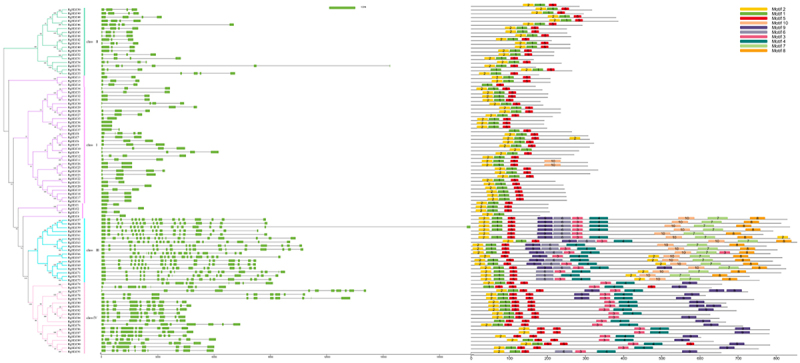
Structure and Motifs of *RgHDZ*. The exons are represented by green boxes, the introns are represented by lines. The motifs, numbers 1–10, are displayed in different colored boxes.

Using RgHDZ proteins sequences to construct a phylogenetic tree to study the evolutionary relationship among members of the family, the results are consistent with the phylogenetic analysis. According to the results of annotation, the structure map of *RgHDZ* genes was constructed. The results showed that the number of exons of RgHDZ was among 2–19 and the number of introns was between 1 and 18. And we found that subfamily III has the largest number of exons and introns, the number of exons is 16–18, and the number of introns is 16–19 (Figure 2). To further study the origin and evolutionary pattern of RgHDZs, their amino acid sequences were subjected to the MEME tool and a total of 12 conserved motifs were identified. The identified motifs ranged from 6 to 200 amino acids in length motif 1 and 2, corresponding to the homeobox domain, and motif 5, corresponding to the LZ domain, were in common among all of the RgHDZs (SupplementaryFigure 2). Motifs 3, 4 and 6, which correspond to START domain, were shared in group III and group IV, while motif 8, which correspond to MEKHLA domain, were present in group III but absent in group IV.

- *Glutinosa* is an autotetraploid (4 n = 56), the difference between each set of chromosomes is very small and it is difficult to distinguish the two sets of chromosomes using Hi-C. Hence, only one set of the genome size was mounted to the chromosome level.^2^The location information shows that the RgHDZ gene family is unevenly distributed on the chromosomes of *R. glutinosa*. There are 43 genes distributed on 13 chromosomes. The number of *RgHDZ* genes on chromosomes 6 and 7 is the largest, with 14 in total, while only one *RgHDZ* gene distributed on chromosomes 8, 11, 12 and 13. (Supplementary table 1).

### 2.4. Cis-acting element prediction of RgHDZ

The promoter sequence of the *RgHDZ* genes (1.5 kb upstream of 5ʹUTR) was submitted to the PlantCARE database for cis-elements prediction, and 72 cis-acting elements were identified. As shown in Supplementary figure 2, all *RgHDZ* genes contain 2 conventional promoter elements (TATA-box and CAAT-box). The cis-acting elements frequently predicted in most *RgHDZ* genes mainly fall into four categories: light-responsive elements, shedding Acid response element, anaerobic inducing element and MeJA response element. Among them, there are up to 24 types of light-responsive elements (3-AF1 binding site, AAAC-motif, ACA-motif, ACE, etc.). In addition, there were some that appear less frequently and were only predicted in a few *RgHDZ* genes, such as gibberellin response (GARE-motif, P-box, and TATA-box), salicylic acid response (TCA, SARE), auxin Elements (AuxRE, AuxRR-core), circadian elements (circadian), endosperm expression (AACA-motif, GCN4-motif), and a cis-element (MBSI) is involved in the MYB binding of flavonoid biosynthesis gene regulation Site.

### 2.5. Synteny analysis of RgHDZ genes

In order to clarify the underlying evolutionary mechanism of the *R. glutinosa RgHDZ* gene family, we studied its replication events and the results showed that some large fragments of repetitions were detected on the chromosomes of *R. glutinosa*. These homologous gene pairs were (*RgHDZ4:5, RgHDZ13:15, RgHDZ17:21, RgHDZ19:22, RgHDZ22:21, RgHDZ28:34, RgHDZ46:47, RgHDZ52:56, RgHDZ57:61, RgHDZ58:59, RgHDZ63:58, RgHDZ63:57, RgHDZ63:56, RgHDZ63:64, RgHDZ65:64, RgHDZ66:67, RgHDZ73:69, RgHDZ78:79, RgHDZ90:91, RgHDZ87:92*). The results indicated that *RgHDZ* genes may be amplified by gene duplication.

In order to clarify the role of selection pressure in the evolutionary species of RgHDZ family, the synonymous substitutions (Ks), the nonsynonymous substitution rate (Ka) and their ratio (Ka/Ks) of RgHDZ homologous genes were analyzed using TBtools. Results The Ka/Ks of the 19 pairs of homologous genes were all far less than 1 (Table 2), indicating that the RgHDZ family has undergone strong purification selection during the evolution process

**Table 2.** Ka/Ks value of *RgHDZ* genes.

Gene1	Gene 2	Ka	Ks	Ka/Ks	Effective Len
RgHDZ82	RgHDZ84	0.031815099	0.911474422	0.034905093	2208
RgHDZ87	RgHDZ92	0.285381483	4.269785317	0.066837431	2280
RgHDZ57	RgHDZ63	0.155438834	1.632788878	0.095198366	2331
RgHDZ58	RgHDZ63	0.162955353	1.552813503	0.104941999	1875
RgHDZ64	RgHDZ65	0.005650542	0.05316158	0.106289963	2169
RgHDZ21	RgHDZ22	0.104849738	0.973744191	0.107676881	2247
RgHDZ69	RgHDZ73	0.07059876	0.637104775	0.110811852	2163
RgHDZ15	RgHDZ13	0.144788687	1.016077406	0.142497693	456
RgHDZ21	RgHDZ17	0.308860752	2.148077873	0.143784709	495
RgHDZ65	RgHDZ63	0.070113306	0.476610145	0.147108296	1764
RgHDZ64	RgHDZ63	0.072924604	0.470771389	0.154904494	1875
RgHDZ90	RgHDZ91	0.117034593	0.733740695	0.159504023	1080
RgHDZ34	RgHDZ28	0.198614981	0.960322645	0.206821095	2127
RgHDZ56	RgHDZ52	0.169375644	0.55009578	0.307902097	609
RgHDZ47	RgHDZ46	0.286907364	0.862594367	0.332609828	2376
RgHDZ59	RgHDZ58	0.026793591	0.057439226	0.466468516	2295
RgHDZ61	RgHDZ57	0.045194394	0.077616731	0.582276436	2244
RgHDZ78	RgHDZ79	0.060944292	0.095438714	0.638569919	369
RgHDZ61	RgHDZ58	0.047067168	0.060392826	0.779350318	645

### 2.6. Prediction of protein–protein interaction network

**Figure 3.**
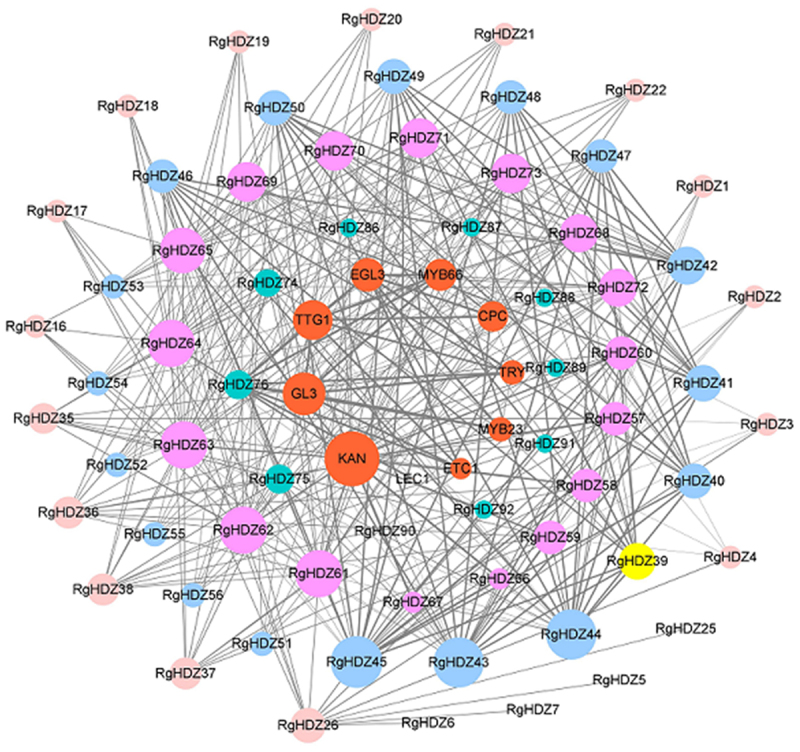
RgHDZ proteins interaction network diagram according to the orthologs in Arabidopsis. The Orange ovals represent the genes interacting with RgHDZ genes.

In order to further understand the interaction of pepper RgHDZ proteins, an interactive network based on *Arabidopsis* orthodoxy was established using STRING. As shown in Figure 3, except for 27 RgHDZs that have not been queried for their interacting proteins, all other RgHDZs have interacting proteins. Among them, the interacting proteins that do not belong to the HD-Zip proteins are KAN (KANADI), GL3 (GLABRA 3), TTG1 (TRANSPARENT TESTA GLABRA 1), EGL3 (ENHANCER OF GLABRA 3), MYB66, CPC (CAPRICE), TRY (TRIPTYCHON), MYB23, and ETC1 (ENHANCER OF TRY AND CPC 1). In interacting proteins networks, the largest hub is KAN proteins (Figure 3). Surprisingly, all class III HD-Zip of *R. glutinosa* interact with KAN. The class III HD-Zip and KANADI genes comprise a genetic system that patterns abaxial-adaxial polarity in lateral organs produced from the apical meristem.^33^ Besides, we found that GL3, TTG1, EGL3, MYB66, CPC, TRY, MYB23, and ETC1 are all related to the development of plant epidermis. Such as GL3 and EGL3 participate in an intercellular regulatory circuit that controls cell patterning in the *Arabidopsis* root epidermis.^34^ In addition, MYB66 and CPC not only participate in the development of *Arabidopsis* trichomes, but also regulate the biosynthesis of anthocyanins.^35–38^ We found that there is a close interaction between II and III class HD-Zip proteins, which may be related to the fact that HD-ZipII and III binding sites share the same core sequence [AAT(G/C)ATT], thus suggesting that members of the two protein families may regulate common target genes.^39^ It is speculated that RgHDZ is involved in the growth and development of *R. glutinosa* and the process of adversity stress through interaction protein analysis.

### 2.7. Analysis of miRNA regulating RgHDZ genes

To further analyze the biological functions of *RgHDZ*,we predicted the miRNA that may regulate the HD-Zip genes of *R. glutinosa*. Results a total of 498 miRNA were predicted to be distributed in 93 families. We found that the class I HD-Zip genes are mainly regulated by miR169 and miR159. Research has shown that the involvement of ma-miR169a (*Musa acuminata* L.) and ma-miR169b in the banana response to Foc4 (*Fusarium oxysporumf*. sp. cubense tropical race 4).^40^ Under drought stress, the differential expression of miRNA regulates the expression of their target genes, resulting in multiple responses of physiological and biochemical pathways relative to drought tolerance of maize.^41^ Several studies have shown that miR169, miR159 had the contributions to plant response toward drought, salinity, cold, and heat, respectively.^42^ This corresponds to the biological function of the aforementioned HD-ZipI subfamily in response to abiotic stress. After prediction, it was found that there are 34 miRNA that regulate HD-ZipII genes, mainly distributed in 10 families: miR4221, miR6459, miR156, miR1072, miR169, miR5139, miR159. The miR6459 is a female characteristic expression gene.^43^The alterations of miR5139, contributed to the attenuation of Cd translocation into the shoot of QLQ.^44^In addition, we found that miR169 and miR159 family genes related to plant abiotic stress. The most number of miRNA were predicted of class III HD-Zip, 272 are distributed in 45 families, including miR166, miR165, miR5168, miR8029, miR447, miR172, etc. The most important ones are miR166 and miR165 families with similar biological functions. Studies have shown that the main target genes of miR166 and miR165 is HD-ZipIII, which regulates the response of target genes to plant stress through differential expression.^41,45^ It has been predicted that 101 miRNA regulating HD-ZipIV genes are distributed in 34 families, mainly miR9671, miR5651, miR7731, miR1149.2, miR9735, miR6169, miR7520, miR2619, miR5260, miR6222, miR482. Except for cellulose hydrolysis, all other cell wall degradation modules are predicted to be targeted by miR6222.^46^ SlymiR482e‐3p mediates tomato wilt disease by modulating ethylene response pathway.^47^

**Figure 4.**
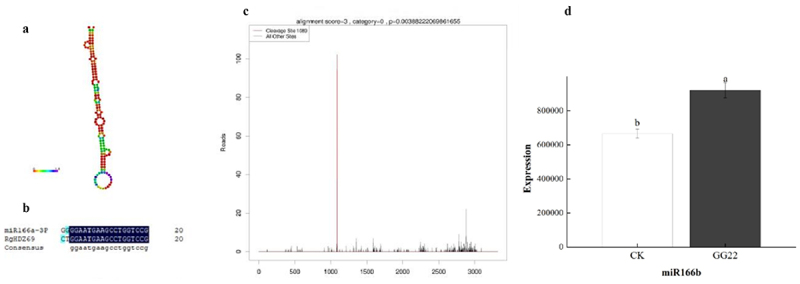
Multiple sequence alignment and t-plot. (a) Predicted secondary structure of MiR166; (b) Alignment of the predicted MREs (miRNA response elements) sequences compared with the miR166 reverse complementary sequences; (c)miR166b slicing RgHDZ69 at nt 1089. (d) Expression of miR166.

It is known that miRNA can inhibit translation or degradation through binding sites (miRNA response elements; MREs) and mRNA complementary pairing.^48^ As shown in Figure 4 -b, miR166-3p and HD-ZipIII gene sequences is reversed at this position complementary, indicating that *R. glutinosa*HD-ZipIII genes can be translationally inhibited or degraded by miR166-3p.

**Figure 5.**
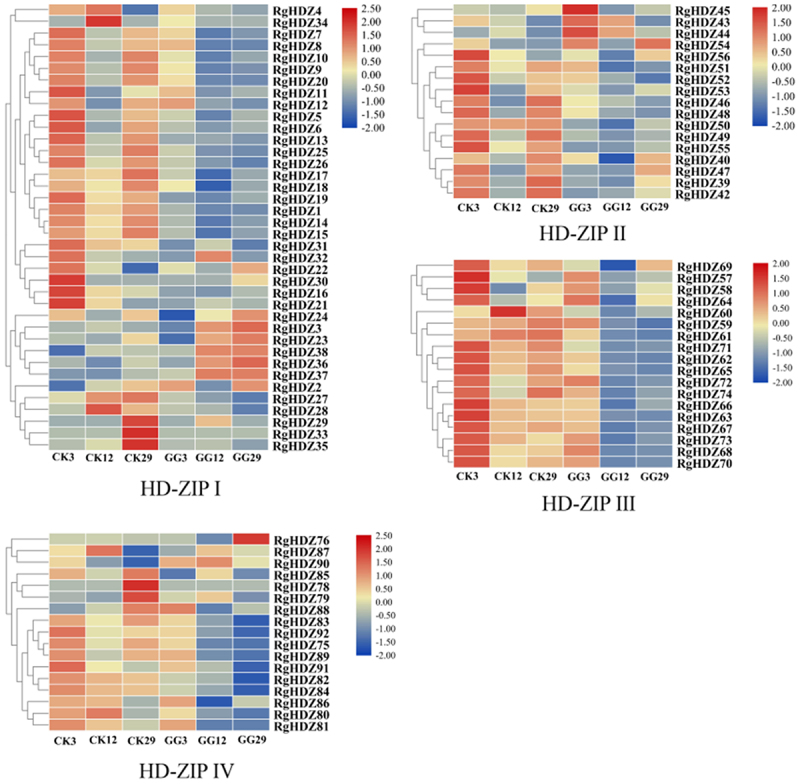
Relative expression of *RgHDZ* after induction of endophytic fungus GG22.

Through the combined analysis of *R. glutinosa* miRNA (PRJNA781675), transcriptome (PRJNA781424), and degradation (PRJNA785421) sequencing. We found that RgHDZ69 was cleaved by miR166a (Figure 4 -c) during the infection process of *R. glutinosa* endophytic fungus GG22 to participate in the regulation of the biological stress process of*R. glutinosa*. Moreover, we found that the change trend of miR166 expression and *RgHDZ69* were mutually antagonistic after GG22 infection (Figure 4 -d; Figure 5).

### 2.8. Expression pattern analysis of RgHDZ in response to endophytic fungi GG22 infection

In order to explore the response of *R. glutinosa* HD-Zip gene in biotic stress. Tissue culture seedlings of *R. glutinosa* that was co-cultured with endophytic fungi GG22 for 3, 12, 29 days, another group was not co-cultured with GG22 were used as materials, named GG3, GG12, GG29 and CK3, CK12, CK29, respectively, for transcriptome sequencing. Set three biological replicates for each group, and analyze and process the data obtained after sequencing.

According to the transcriptome datas, the expression levels of the 92 *RgHDZ* genes were, generally, up or down-regulated expression after infection by the endophytic fungi GG22 (Figure 5). According to statistics, the expression of the 50/92 *RgHDZ* genes were down-regulated during the three sampling periods, and the performance of the class III and IV *RgHDZ* genes were particularly obvious. The differential expression of the group indicates that the HD-Zip family genes may be involved in the regulation of the stress response of *R. glutinosa*. The functions of homologous genes are known to be similar. In order to explore more different functions of RgHDZ genes, non-homologous genes were selected for experiments according to the results of phylogenetic tree and homologous gene analysis. For the purpose of studying whether it is involved in the abiotic stress response of *R. glutinosa*, 11 differentially expressed genes were selected from different branches of each subfamily (Figure 1), and qRT-PCR was used to detect their effects under drought, waterlogging, high temperature, low temperature, and salt stress to analyze the expression pattern of *RgHDZ* genes in response to abiotic stresses.

### 2.9. Expression pattern analysis of RgHDZgenes in response to high and low temperature stresses

**Figure 6.**
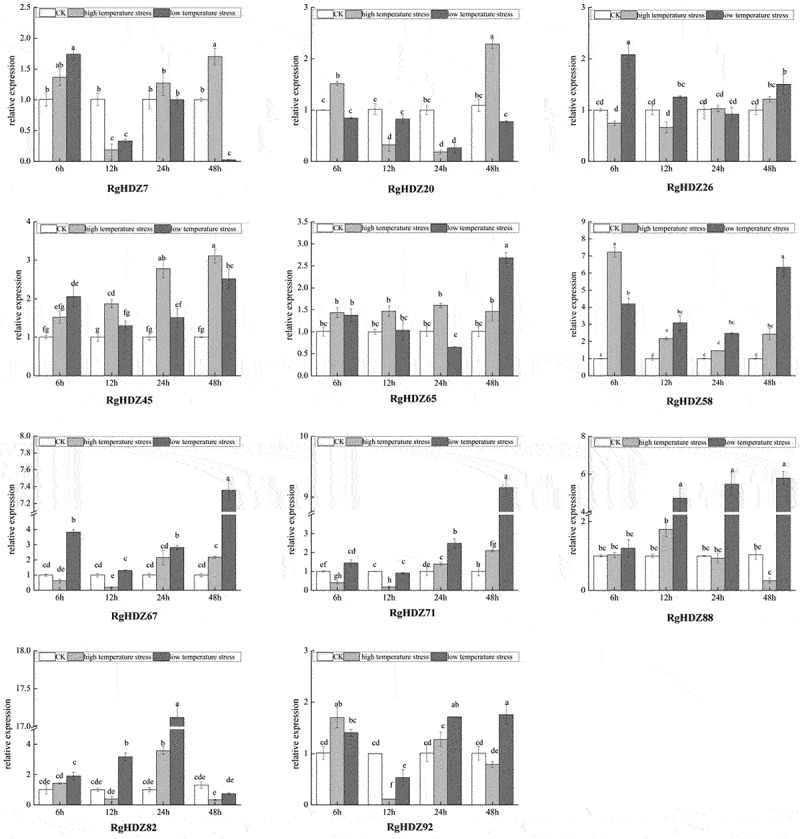
Expression of *RgHDZ*genes in response to high and low temperature stresses. Total RNAs from the stressed samples were extracted from the third leaf of each plant. All data represent the mean ± standard deviation of three independent experiments. abcde indicate significant differences than control group (Student’s t-test; P < .05).

The expression levels of the selected *RgHDZ* genes were changed in response to high and low-temperature stresses, but some differences were present among these genes. Under high-temperature stress, the expression levels of *RgHD*Z45 and *RgHDZ65* were significantly up-regulated through all the time points, while *RgHDZ45* and 65 were up-regulated through all the time points in low-temperature stresses, with the highest expression levels at 48 h. In addition, some *RgHDZ* genes exhibited similar expression profiles under high- and low-temperature stresses, such as *RgHDZ58, RgHDZ82*and *RgHDZ92*. However, some *RgHDZ* genes displayed an obvious decrease in expression under high- and low-temperature stresses at certain time points, such as *RgHDZ7* and *RgHDZ20* at 12 h and 24 h, respectively (Figure 6).

### 2.10. Expression pattern analysis of RgHDZ genes in response to salinity stresses

**Figure 7.**
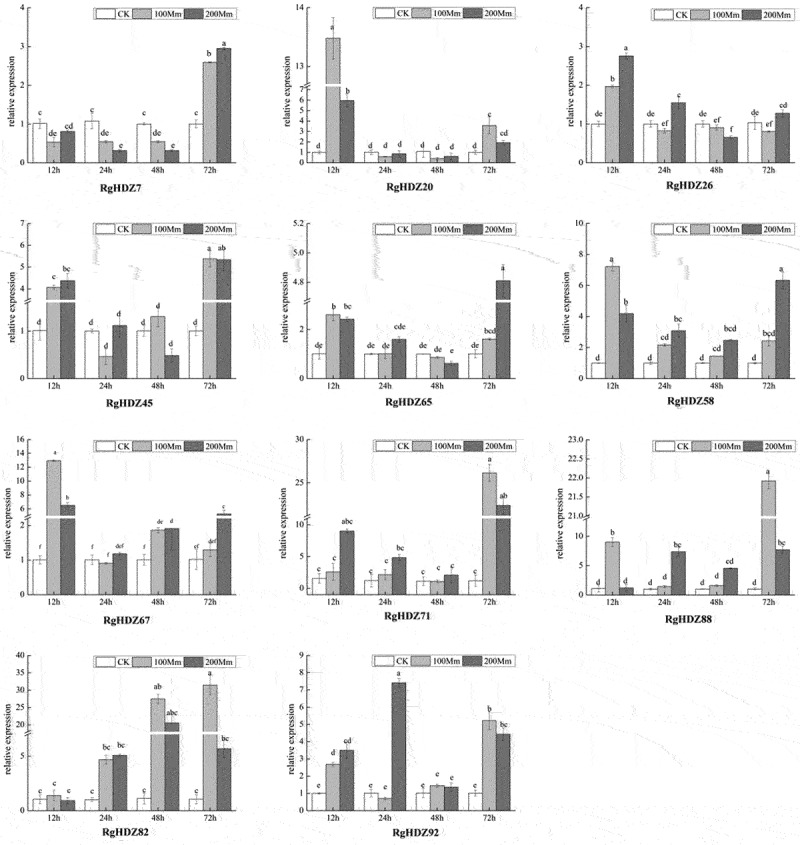
Expression of *RgHDZ* genes in response to salinity stresses. Total RNAs from the stressed samples were extracted from the third leaf of each plant. All data represent the mean ± standard deviation of three independent experiments. abcde indicate significant differences than control group (Student’s t-test; P < .05).

In order to identify the role of *RgHDZ* genes under salt stress, the expression patterns of the above 11 genes that respond to temperature stress were analyzed their expression patterns by qRT-PCR. Overall, the expression levels of all the selected genes were significantly changed in response to salinity stress, but some differences were present among these genes. Under salinity stress, the expression levels of *RgHDZ58* was up-regulated through all the time points, while *RgHDZ20* and 67 were induced at certain time points, with the highest expression levels at 12 h, respectively. However, other *RgHDZ* genes displayed an obvious decrease in expression under salinity stress at certain time points. Under salinity stress, most of the selected *RgHDZ* genes showed strong up-regulation in expression at some of the time points, while *RgHDZ7, 20, 26, 45, 65, 58*, and *67* kept low transcription levels in some time points (Figure 7).

### 2.11. Expression pattern analysis of RgHDZ genes in response to drought and waterlogging stresses

**Figure 8.**
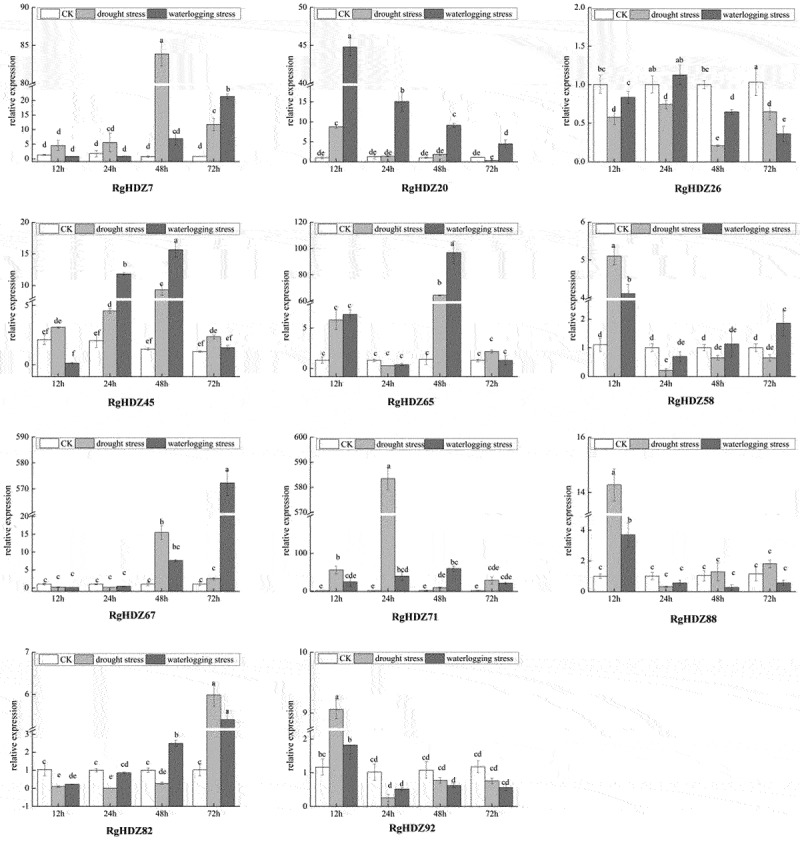
Expression of *RgHDZ* genes in response to drought and Waterlogging stresses.Total RNAs from the stressed samples were extracted from the third leaf of each plant. All data represent the mean ± standard deviation of three independent experiments. abcde indicate significant differences than control group (Student’s t-test; P < .05).

It was observed that most of the *RgHDZ* genes were differentially affected under drought and waterlogging stresses. For example, two *RgHDZ* genes, *RgHDZ7* and *RgHDZ45*, were up-regulated under drought stress, with their expression markedly increasing at 12 h, and peaking at 48 h (Figure11). *RgHDZ20* was up-regulated under waterlogging stress, with their expression markedly increasing at 12 h to 48 h, and peaking at 12 h. Some *RgHDZ* genes, such as *RgHDZ20, RgHDZ58, RgHDZ88*, and *RgHDZ92*, were also up-regulated under stress, but their expression levels were peaked at 12 h, implying their roles in early response to drought and Waterlogging stress. However, some *RgHDZ* genes, such as *RgHDZ20, RgHDZ26, RgHDZ45, RgHDZ65, RgHDZ58, RgHDZ88, RgHDZ82*, and *RgHDZ92*, exhibited similar expression profiles after drought and Waterlogging stresses. Moreover, it was showed that the response of *RgHDZ* genes to waterlogging is lagging to drought, such as *RgHDZ7* and *RgHDZ67* (Figure 8).

### 2.12. Expression pattern analysis of RgHDZ genes in response to biotic stresses

Different from GG22, *Fusarium solani*, an important plant pathogen can cause root rot in plants, which mainly infects the rhizomes of the host, causing root rot and blocking the ducts, and ultimately the host death.

**Figure 9.**
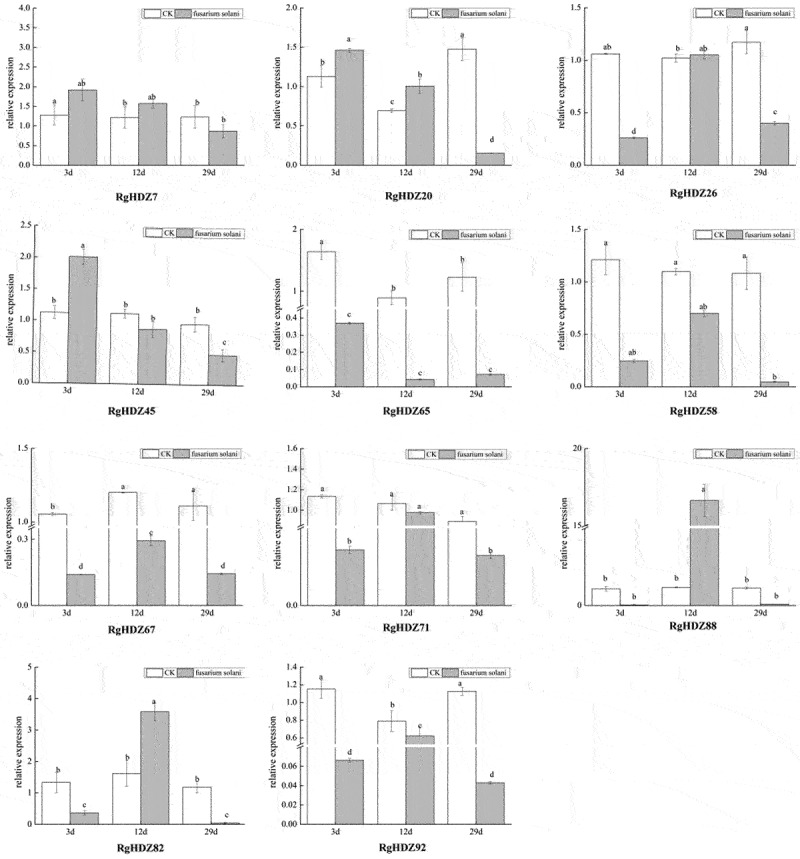
Expression of *RgHDZ* genes in response to *Fusarium solani* stresses. Total RNAs from the stressed samples were extracted from the third leaf of each plant. All data represent the mean ± standard deviation of three independent experiments. abcde indicate significant differences than control group (Student’s t-test; P < .05).

In this experiment, the change of *RgHDZ*genes expression in the tissue cultured seedlings of *R. glutinosa* co-cultured with *Fusarium solani* was investigated to study the role of *RgHDZ* genes in fungi stress of *R. glutinosa*. Expression of *RgHDZ* genes change significantly under pathogenic fungus stress, but it is generally at a low level (Figure 9). Overall, the expression levels of *RgHDZ* genes were significantly down-regulated in response to pathogenic fungus stress, but *RgHDZ20, RgHDZ45, RgHDZ82*, and *RgHDZ88* up-regulated at certain time points, with the highest expression levels at 3 d, 3 d, 12 d, and 12 d, respectively. It can also be found from the results that the class III HD-Zip genes *RgHDZ65, RgHDZ58, RgHDZ67*, and *RgHDZ71* were down-regulated in all sampling periods after Fusarium solani infection, which is similar with nonpathogenic fungus GG22 infection. Moreover, *RgHDZ7* and *RgHDZ20* exhibited similar expression profiles after pathogenic fungus stress. Taken together, these results suggested that these genes might play a vital role in response to multiple biotic stresses in *R. glutinosa* (Figure 9).

↑ means at least one time of expression that is up-regulated and there is no down-regulation in any other periods, ↓means at least one time of expression that is down-regulated and there is no up-regulation in any other periods, M means both up- and down-regulation of expression in different periods, – means no significant change in expression in all periods.

As seen in Table 3, all 11 RgHDZ genes were able to respond to a variety of different stress conditions. In general, the majority of RgHDZ gene expressions were up-regulated in response to abiotic stresses like heat, salt, and drought; however, the majority of RgHDZ gene expressions exhibited a decreasing trend in response to pathogenic fungal infestation.

**Table 3.** Statistics of relative expression changes of *RgHDZ* under various stresses.

Gene name	high temperature	low temperature	100 mM Nacl	200 mM Nacl	drought	waterlogging	Fusarium solani
RgHDZ7	M	M	M	M	↑	↑	-
RgHDZ20	M	↓	↑	↑	↑	↑	M
RgHDZ26	-	↑	↑	M	↓	↓	↓
RgHDZ45	↑	↑	↑	↑	↑	↑	M
RgHDZ58	↑	↑	↑	↑	M	↑	↓
RgHDZ65	-	↑	↑	↑	↑	↑	↓
RgHDZ67	↓	↑	↑	↑	↑	↑	↓
RgHDZ71	M	↑	↑	↑	↑	↑	↓
RgHDZ82	↑	↑	↑	-	M	M	M
RgHDZ88	↓	↑	↑	↑	↑	↑	↑
RgHDZ92	↑	M	↑	↑	↑	-	↓

## 3. Discussion

### 3.1. Identification and phylogenetic analysis of HD-Zip TFs in R. glutinosa

*HD-Zip* genes encode a family of plant-specific transcription factors involved in various biological processes in plants. In this study, we identified 92 *HD-Zip* genes from *R. glutinosa* at the Genomic level. The number of HD-Zip members in *R. glutinosa* was nearly twice as much to that of *Arabidopsis*(48),^17^ rice(48),^49^ wheat(46).^25^ Combined with the analysis of collinearity, it was found that it may be due to the replication of large fragments that caused the amplification of the *HD-Zip* genes family in *R. glutinosa*.

At present, more and more evidences show that the *HD-Zip* genes family plays an important role in plant life. For example, AtHB13 was able to confer tolerance to freezing temperatures via the induction of glucanase (GLU and PR2) and chitinase (PR4) proteins,^5^ which could also regulate drought stress together with the target *JUB1* gene.^50,51^ ATHB23 plays role in salt and osmotic stress tolerance in *Arabidopsi*s.^52^ ATHB16 affects the content of ABA in plants by responding to blue light,^52^ ATHB4 and ATHAT3, members of the class II HD-Zip transcription factor, have been shown to play an instrumental role in the responses to shade.^53^ However, REV in *Arabidopsis* is related to the development of leaf polarity and overlaps with PHB and PHV functions.^54^ Many studies have shown that class IV HD-Zip proteins are involved in plant abiotic stress processes, such as over-expression of *AtHDG11* gene confers drought tolerance in plant;^55,56^ *AtPDF2*, the *Arabidopsis* defensin gene, promotes cytoplasmic Cd efflux via chelation, thereby enhancing Cd detoxification and apoplastic accumulation;^57^ According to the phylogenetic tree, collinearity analysis and Ka/Ks analysis, we can speculate that the *RgHDZ* gene is involved in regulating the growth and development of *R. glutinosa* and adversity stress.

### 3.2. RgHDZs protein interaction network

The establishment of protein interaction network is an important method for predicting protein function, which can be predicted based on the protein directly or indirectly interacting with the protein to be tested. As shown in Figure 5, there were interactions between RgHDZs protein members, which jointly regulate the complex life activities of plants. In addition, many proteins that do not belong to the HD-Zip interact with RgHDZs, such as KAN, GL3, TTG1, EGL3, MYB66, CPC, TRY, MYB23, and ETC1. The core protein KAN in the interaction network diagram is related to the development of plant organs,^33,58^ and most of the proteins that interact with KAN belong to the class III RgHDZ protein. According to the literature search, we know that the class III HD-Zip protein is related to the development of plant epidermis, so through interaction analysis, not only helps us understand the protein interaction relationship of RgHDZ, but also can further speculate on its biological function. For example, we can speculate that type III RgHDZ may play an important role in the development of the organs of *R. glutinosa*. Based on the proven biological functions of these proteins, we predict the biological function of the RgHDZs protein to be tested, which may respond to a variety of abiotic stress processes, such as controlling the ABA content in *R. glutinosa* to affect plant resistance ability.

### 3.3. MicroRNA prediction

MiRNA, short 21-nucleotide RNA molecules, play an important role in post-transcriptional regulation of gene expression miRNA prediction of *RgHDZ* genes found that different HD-Zip family genes will be regulated by the same miRNA, and the same target gene will also be regulated by different miRNA. At present, there were more studies on the targeted regulation of miR166/miR165 on HD-ZipIII family genes. The *Arabidopsis* miR166/165 group targets five members of the HD-ZipIII transcription factor (REVOLUTA, PHABULOSA, PHAVOLUTA, CORONA, ATHB15, and ATHB8) functions by cleaving target mRNAs through complementary base pairing.^59,60,^^61^ Furthermore, studies have shown that the differential expression of miR166/165 could regulate the abiotic response of plants.^45^ Kitazumi researched that Inverse relationships have been established between the expression of two salt stress-regulated miRNA (miR166, miR159) and the transcriptional regulators they control (HD-Zip-Phabulosa/Phavoluta and Myb101, respectively) (Kitazumi et al., 2015). Moreover, the expression of miR166, miR159 and miR156 was up-regulated in Cavendish banana roots inoculated with Fusarium oxysporum.^62^ Apparently, miR166 participates in the regulation of a variety of life activities, and also plays an important role in the process of plant stress response. According to the prediction results, we have found that the HD-ZipIII subfamily of *R. glutinosa* was regulated by miR166 and miR165, indicating that HD-ZipIIIs participates in the biotic and abiotic stress process of *R. glutinosa* and is regulated by miR166 and miR165. In addition, the other three subfamily HD-Zip genes have predicted miRNA related to plant stress. All in all, the *RgHDZ* genes may be regulated by miRNA in the process of participating in the stress response.

### 3.4. The expression pattern of RgHDZ genes

The expression of *RgHDZ* genes under biotic and abiotic stresses were tested, and the results clearly verified the hypothesis that it participates in the stress of *R. glutinosa*. (Table 3) Observing the expression of 11 *RgHDZ* genes under different abiotic stresses, it was found that most of the genes after abiotic stress were up-regulated compared to the control group during most of the period. And Compared with temperature and salt stresses, the *RgHDZ* genes had the most significant up-regulation reaction occurred in the drought and waterlogging stress group. This indicates that *RgHDZ* genes may play an important role in the process of *R. glutinosa* coping with drought and waterlogging stress. Related studies in *Arabidopsis* also found that the expression of HD-ZipI subfamily genes *ATHB6* and *ATHB7* were induced by water stress,^63,64^ there were few studies on water-related stress in the III and IV subfamily. Our studies have found that its expression is induced in water-related stress, indicating that it may be involved in abiotic stress related to water in *R. glutinosa*.

Different from abiotic stress expression pattern, that the changes of *RgHDZ* genes expression after infection by *Fusarium solani* was very similar to infection by GG22, compared with the control group, the expression of *RgHDZ* was mainly down-regulated. Among them, *RgHDZ88* and *RgHDZ82* were down-regulated in the three sampling times. Therefore, the *RgHDZ* genes of *R. glutinosa* may mainly respond to the biological stress of fungal infection by down-regulating its expression.

## 4. Materials and methods

1. *Identification of R. glutinosa HD-ZIP transcription factors and analysis of its physical and chemical properties*

The Hidden Markov Model (HMM) profile of the homeodomain (HD) (PF00046) and the leucine zipper (LZ) domain (PF02183) were download from PFAM database (http://pfam.xfam.org/), which employed as queries to search against the Genome database of *R. glutinosa* using the program HMMER (http://hmmer.org/) to obtain all sequences containing the conserved domains of HD and LZ. After removing redundant sequences, the SMART database was used to examine the presence of the HD and LZ domains for each identified candidate. Finally, non-redundant HD-Zip encoding genes were identified in *R. glutinosa*, and denominated as RgHDZ. Using online tools to analyze the physical and chemical properties and secondary structure of HD-Zip, such as ProtParam (http://us.expasy.org/tools/protparam.html), TargetP (http://www.cbs.dtu.dk/services/TargetP/), SOPMA (https://npsa-prabi.ibcp.fr/cgi-bin/npsa_automat.pl?page=npsa_sopma.html), ProtScale (http://web.expasy.org/protscale/), TMHMM Server v.2.0 (http://www.cbs.dtu.dk/services/TMHMM/).

### 4.2. Phylogenetic, exon-intron structure and conserved motif analyses of HD-zip family in R. glutinosa

The protein sequences of HD-Zip from *R. glutinosa* and *Arabidopsis* were used to construct the phylogenetic tree by MEGA 7, using the neighbor-joining (NJ) method with 1000 bootstrap replications. Import the results into online software iTOL (https://itol.embl.de/) for beautification. Use GSDS 2.0 (Gene Structure Display Server) (http://gsds.gao-lab.org/) to determine the intron-exon structure of the *RgHDZ* gene. Conserved motifs present in RgHDZ were identified using MEME (https://meme-suite.org/meme/tools/meme). The following parameters were used: number of repetitions any, maximum number of motifs 12, and the optimum motif widths were constrained to between 6 and 100 residues.

### 4.3. Promoter analysis

The 1500 bp upstream sequences of coding region of *RgHDZ* genes were downloaded from *R. glutinosa* Genome Database. The cis-regulatory elements were identified using online program PlantCARE(http://bioinformatics.psb.ugent.be/webtools/plantcare/html/).

### 4.4. Interaction protein analysis

Interaction protein in RgHDZs were identified using STRING (https://string-db.org/), and used Cytoscape to draw a protein interaction network diagram.

### 4.5. Predict the microRNAs that regulate RgHDZgenes

Using psRNATarget (http://plantgrn.noble.org/psRNATarget/), select the reported *Arabidopsis* transcript and miRNA database, and use the psRNATarget scoring model V2 (2017) default conditions for prediction.

Six groups of *R. glutinosa* tissue cultured seedlings RNA were extracted, and after passing the quality test, the sRNA library was constructed, using the Hiseq 2500 platform for sequencing. Then we processed and analyzed the raw data of sequencing, removed the 3ʹlinker and junk sequence, and obtained clean data; The sequences with a base length of 18–25 nt are reserved for comparison with various RNA databases, such as mRNA, RFam and Repbase database, and filtered. The finally obtained data is valid data for subsequent small RNA data analysis. Compare the valid data with the miRNAs included in miRBase for miRNAs identification.

Perform target gene prediction on the identified miRNAs, and verify the predicted target genes by degradome sequencing. Construction of degradation group library: (1) Capture mRNA with magnetic beads and connect with 3’ and 5’ adaptors, (2) Mixed reverse transcription of Biotinylated Random Primers and mRNA, (3) PCR amplification, after completing the entire library preparation, the constructed library is sequenced with Illumina Hiseq2000/2500, and the sequencing read length is single-ended 1 × 50 bp.

The target gene corresponding to the predicted miRNA is combined with the mRNA in the generated degradation group density file to find the common mRNA, which is the target gene of the miRNA. The peak classification and score of the degradation group are given, and the predicted results are plotted (t-plots).

### 4.6. Expression patterns analysis

#### 4.6.1. Experimental materials

*Fusarium solani*:In the early stage, the laboratory separated and identified the fungi species from the leaves of *R. glutinosa*, PDA culture medium 5 to 7 days activation standby.

Transfer the *R. glutinosa* tissue culture seedlings grown in the MS medium to a plant height of 3–5 cm to the vermiculite medium to adapt to grow for 3 days, and then subjected to high (40°C), low (0°C) temperature, drought, waterlogging, and salt stress (100 mM, 200 mM). In addition, the biological stress group was inoculated with a 5 mm × 5 mm piece of *Fusarium solani*.Three biological replicates were performed for each experiment, and the sample of each replicate from 15 shares plants.

#### 4.6.2. *Expression characteristics of* RgHDZ *after GG22 infection*

To analyze the expression profiles of *RgHDZ* genes in response to GG22 (*R. glutinosa* endophytic fungus) stress, the transcriptome data were used. Heatmaps were visualized with TB tools.^65^

4.6.3. qRT-PCR

In order to explore the role of *RgHDZ* genes in responding to biotic and abiotic stresses, 11 differentially expressed genes were selected from different branches of each subfamily of *R. glutinosa* HD-Zip phylogenetic tree. The relative expression of *RgHDZ* genes in the tissue cultured seedlings of *R. glutinosa* under various abiotic and biotic stresses treatments were analyzed by qRT-PCR. Significant up- and down-regulated genes were determined as *p* < .05 using T test.

The total RNA of *R. glutinosa* was extracted with TRIzon (Beijing ComWin Biotech). BeyoRTTM III First Strand cDNA Synthesis Kit (Beyotime Biotechnology, Shanghai, China) was used to synthesize the first cDNA strand, qRT-PCR primers of *RgHDZ* and a reference gene *TIP41* are listed in Supplementary Table 3. The specific methods for qRT-PCR were performed according to Wang et al.^66^

## 5. Conclusions

We identified 92 HD-Zip family genes from *R. glutinosa*. After bioinformatics and expression pattern analysis, we found that the HD-Zip family genes of *R. glutinosa* may not only regulate the growth and development of plants, but also participate in the biotic and abiotic stress process. The research showed that *RgHDZ* may respond to various stresses by down (or up)-regulating its expression. Since, the entire growth and development stage of *R. glutinosa*, especially during the root expansion period, is vulnerable to various biotic and abiotic stresses, which greatly limits its yield and quality. This study analyzed the expression patterns of HD-Zip family genes in various coercions. Provide the theoretical basis for the cultivation of *R. glutinosa* resistant varieties.

## Data Availability

All data supporting the findings of this study are available within the paper and within its supplementary data published online.
